# Changes in CT-Based Morphological Features of the Kidney with Declining Glomerular Filtration Rate in Chronic Kidney Disease

**DOI:** 10.3390/diagnostics13030402

**Published:** 2023-01-22

**Authors:** Yoon Ho Choi, Seongho Jo, Ro Woon Lee, Ji-Eun Kim, Jin Hyuk Paek, Byoungje Kim, Soo-Yong Shin, Seun Deuk Hwang, Seoung Woo Lee, Joon Ho Song, Kipyo Kim

**Affiliations:** 1 Department of Artificial Intelligence and Informatics, Mayo Clinic, Jacksonville, FL 32224, USA; 2 Department of Digital Health, SAIHST, Sungkyunkwan University, Seoul 06355, Republic of Korea; 3 Division of Nephrology and Hypertension, Department of Internal Medicine, Inha University Hospital, Inha University College of Medicine, Incheon 22332, Republic of Korea; 4 Department of Radiology, Inha University College of Medicine, Incheon 22332, Republic of Korea; 5 Department of Internal Medicine, Keimyung University School of Medicine, Daegu 42601, Republic of Korea; 6 Department of Radiology, Keimyung University School of Medicine, Daegu 42601, Republic of Korea

**Keywords:** chronic kidney disease, kidney shape, glomerular filtration rate, radiomics

## Abstract

Chronic kidney disease (CKD) progression involves morphological changes in the kidney, such as decreased length and thickness, with associated histopathological alterations. However, the relationship between morphological changes in the kidneys and glomerular filtration rate (GFR) has not been quantitatively and comprehensively evaluated. We evaluated the three-dimensional size and shape of the kidney using computed tomography (CT)-derived features in relation to kidney function. We included 257 patients aged ≥18 years who underwent non-contrast abdominal CT at the Inha University Hospital. The features were quantified using predefined algorithms in the pyRadiomics package after kidney segmentation. All features, except for flatness, significantly correlated with estimated GFR (eGFR). The surface-area-to-volume ratio (SVR) showed the strongest negative correlation (r = −0.75, *p* < 0.0001). Kidney size features, such as volume and diameter, showed moderate to high positive correlations; other morphological features showed low to moderate correlations. The calculated area under the receiver operating characteristic (ROC) curve (AUC) for different features ranged from 0.51 (for elongation) to 0.86 (for SVR) for different eGFR thresholds. Diabetes patients had weaker correlations between the studied features and eGFR and showed less bumpy surfaces in three-dimensional visualization. We identified alterations in the CKD kidney based on various three-dimensional shape and size features, with their potential diagnostic value.

## 1. Introduction

Chronic kidney disease (CKD) is a leading cause of mortality and morbidity worldwide. The global prevalence of CKD was estimated to be 9.1% in 2017 and continues to increase due to population aging [1]. CKD also serves as a major risk factor for cardiovascular events and end-stage kidney disease [2].

CKD progression involves both micro- and macrostructural morphological changes in the kidneys. Microstructural changes reflect histopathological processes, such as glomerular sclerosis and interstitial fibrosis. Conventionally, histological findings are described according to different renal compartments in a semi-quantitative manner [3,4]. Many studies have reported associations of histological findings or grades with kidney function and outcomes [5,6]. In this context, histological grading has been successfully utilized for risk stratification in CKD [7,8]. In contrast, macrostructural changes in CKD have been mainly described using simple measures, such as kidney length and cortical thickness, through kidney imaging.

Given that CKD is defined as persistent structural or functional abnormalities of the kidney for ≥3 months [9], kidney imaging techniques, such as ultrasound (US) and computed tomography (CT), are widely used for the evaluation of CKD. Visual assessment of the kidney by ultrasound or CT scan shows morphological changes in the kidney of patients with CKD. Decreased kidney length and cortical thickness identified on kidney imaging correlate with reduced glomerular filtration rate (GFR). Nonetheless, kidney length and thickness both lack sensitivity and specificity as diagnostic markers for CKD. Moreover, the methodology used to acquire these measures of the kidneys is not standardized, and a diagnostic threshold has not been determined. Other combined parameters, such as kidney length-to-height ratio or renal shape index (RSI), have been suggested as alternatives [10,11]. However, these methods are still simple and lack accuracy, with some issues around reproducibility and reliability.

Indeed, clinicians empirically know the morphological changes, such as the shape and size of the kidney, in patients with CKD. However, geometrical alterations in the kidney have not been fully evaluated quantitatively and comprehensively. In this study, we aimed to analyze the CT-derived, radiomics-based morphological features of the kidney in relation to kidney function and to assess the diagnostic value of these features for CKD.

## 2. Materials and Methods

### 2.1. Study Design

We retrospectively enrolled patients aged ≥18 years who had undergone non-contrast abdominal CT at Inha University Hospital (INUH) between 1 January 2015 and 31 March 2019. We excluded (1) patients without serum creatinine values measured in steady state within three months before and after CT examination, (2) those who were diagnosed with acute kidney injury or receiving renal replacement therapy (hemodialysis, peritoneal dialysis, and kidney transplantation), (3) those who had a CT scan of poor image quality, and (4) those who had hydronephrosis, large cysts, or a single kidney. Demographic data, including age, sex, height, body weight, and past medical history, were collected from the electronic health records of the INUH. Estimated glomerular filtration rate (eGFR) was calculated using the Chronic Kidney Disease Epidemiology Collaboration (CKD-EPI) equation [12]. We quantified 17 different morphological features from the selected CT images and evaluated the associations between feature values and eGFR.

### 2.2. Image Preprocessing and Feature Extraction

Three-dimensional kidney segmentation was performed using a semi-automatic tool (AVIEW Research; Coreline Soft, Seoul, Republic of Korea) by two experienced radiologists, followed by manual modification and confirmation by consensus of them. All CT images and their kidney segmentations were resampled to a voxel size of 1 × 1 × 1 mm using B-spline and nearest-neighbor interpolation. Image preprocessing was performed using SimpleITK (version 2.1.1; Kitware Inc., Clifton Park, NY, USA). Evaluated features were quantified according to the definitions implemented in the pyRadiomics package (version 3.0.1; https://pyradiomics.readthedocs.io, accessed on 30 November 2022). The morphological features used were mesh volume, voxel volume, surface area, surface-area-to-volume ratio (SVR), sphericity, spherical disproportion, compactness 1 and 2, maximum three-dimensional (3D) diameter, maximum two-dimensional (2D) diameter (axial/sagittal/coronal), major/minor axis length, least axis length, elongation, and flatness. The mean value of each feature for the right and left kidneys was used in the analysis. Detailed definitions and explanations of the features are provided in Supplementary Table S1 [13,14]. In addition, the average kidney shape was visualized to assess the gross morphological changes associated with decreased GFR. All images of left kidneys within the same GFR ranges were centered, and the kidney masks were averaged and binarized based on 0.5 to visualize the representative mask images using the itkwidget package. This study was conducted in accordance with the World Medical Association Declaration of Helsinki and approved by the Institutional Review Board of INUH (IRB No. 2020-10-009).

### 2.3. Statistical Analyses

The characteristics of participants were presented as the mean ± standard deviation or median and interquartile range based on the normality test for continuous variables and as counts and percentages for categorical variables. Pearson analysis was performed to explore the correlation between shape features and eGFR. Since morphological features, such as kidney volume and length, depend on the individual’s body size, correlation analysis was performed with eGFR deindexed by multiplying the individual’s BSA/1.73 m^2^. A clustered heat map was created to examine the correlation patterns of the features. Additionally, we performed receiver operating characteristic (ROC) curve analyses to examine the discriminant ability of each shape feature for different eGFR thresholds for CKD stages (30, 45, and 60 mL/min/1.73 m^2^). Univariable and multivariable linear regression analyses were performed to identify the determinant factors of the SVR. Statistically significant variables in the univariate analysis were used in the multivariate analysis. *p*-values of <0.05 were considered statistically significant. All analyses were performed using Python software (Python Software Foundation, version 3.9.7) and R software, version 4.1.2. (R Foundation for Statistical Computing, Vienna, Austria).

## 3. Results

### 3.1. Patient Characteristics and Feature Summary

In total, 257 patients were included in the analysis. The mean age was 68 years, 55.6% were men, 39% had diabetes, mean eGFR was 57.2 mL/min/1.73 m^2^, and 59.5% had eGFR < 60 mL/min/1.73 m^2^ (Table 1). Our study population included 163 patients with CKD (63.4%); the causes of CKD were diabetic nephropathy (23.3%), hypertensive nephropathy (20.6%), biopsy-proven glomerulonephritis (3.1%), and others (16.4%).

The morphological features of the participants are summarized in Supplementary Table S2. The mean kidney volume estimated using the 3D mesh was approximately 124,412 mm^3^, and the surface area was 24,639 mm^2^. The mean maximal diameter ranged from 64.0 mm (2D axial) to 107.0 mm (3D). Assuming an ellipsoidal kidney shape, mean major axis length was measured as 99.5 mm, mean minor axis length was measured as 54.5 mm, and mean least axis length was measured as 45.1 mm.

### 3.2. Correlation Analysis

**Figure 1:**
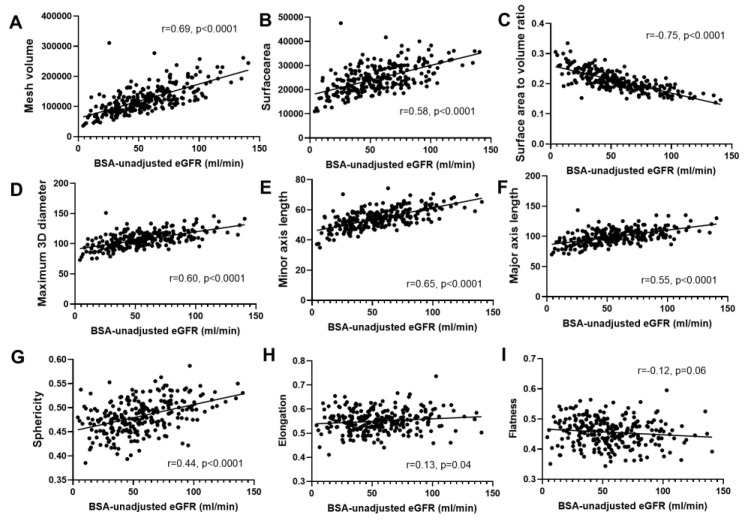
Correlations between morphological features and BSA-unadjusted eGFR. (**A**) Mesh volume. (**B**) Surface area. (**C**) Surface-area-to-volume ratio. (**D**) Maximum 3D diameter. (**E**) Minor axis length. (**F**) Major axis length. (**G**) Sphericity. (**H**) Elongation. (**I**) Flatness.

All extracted features other than flatness revealed a significant correlation with eGFR (Table 2). The Pearson coefficients of correlation ranged from −0.75 to 0.69. The SVR displayed the strongest negative correlation (r = −0.75, *p* < 0.0001), and the kidney volume (mesh and voxel volume) showed the strongest positive correlation (r = 0.69, *p* < 0.0001). The mesh and voxel volumes had nearly the same correlation coefficients. Sphericity and major axis length showed modest correlations (r = 0.55 and 0.44, respectively). Elongation and flatness showed weak correlations (r = 0.13 and −0.12, respectively). Of the diameter features, minor axis length was most strongly correlated with eGFR, followed by maximum 3D and 2D diameters (from coronal view). The major axis length, which is conventionally used as the kidney length, showed a relatively lower correlation than the minor axis length. These findings suggest that the minor axis length could be more useful than the other length markers. The minor axis length is defined as the second-largest axis length of an ellipsoid, which represents the width of the kidney from medial to lateral. The correlation between the representative features and kidney function is shown in Figure 1. In patients with diabetes, the overall correlation with eGFR was similar but slightly lower than that in patients without diabetes (Table 2). The kidney volume showed the highest correlation with eGFR in diabetic patients, but elongation and flatness had no correlation with eGFR. Additionally, we examined the influence of covariates, such as age, sex, and BMI, on the correlation between morphological features and eGFR (Supplementary Tables S3–S5). As a result, morphological features showed a higher correlation with eGFR in female and younger patients.

**Figure 2:**
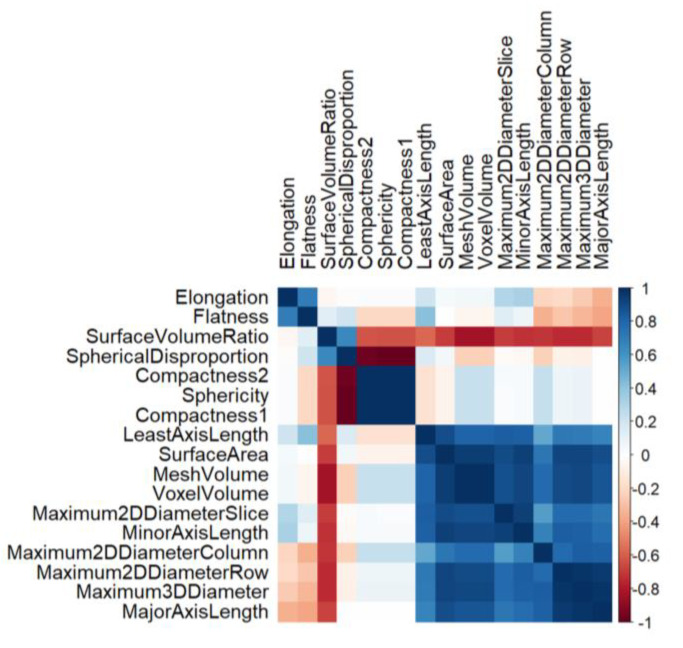
Clustered correlation matrix of the morphological features.

The correlation matrix revealed relationships between morphological features (Figure 2). Three distinct feature clusters were identified. Kidney volume, diameter, surface area, and the size parameters of the kidney were positively clustered. The elongation and flatness, which are 2D-shape features with little relation to the kidney size, were gathered in the upper-left corner of the correlation matrix. The SVR, sphericity, spherical disproportion, and compactness were also clustered together, which are defined using different formulas for the two variables (surface area and kidney volume). Notably, unlike other shape features, only the SVR had a negative correlation with the size features.

### 3.3. Diagnostic Value of Features and 3D Visualization

**Figure 3:**
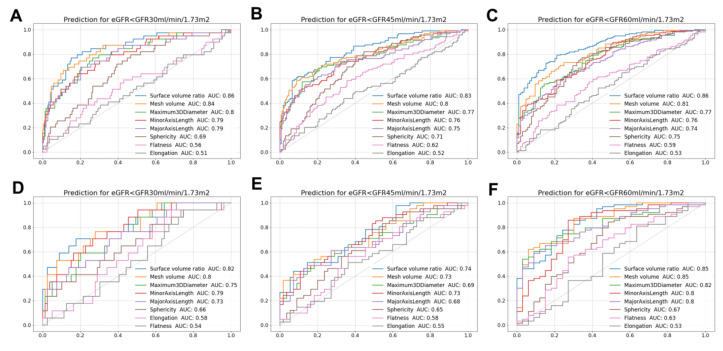
ROC curves of the morphological features for different eGFR thresholds. ROC curves in total participants for eGFR (**A**) <30 mL/min/1.73 m^2^, (**B**) <45 mL/min/1.73 m^2^, and (**C**) <60 mL/min/1.73 m^2^. ROC curves in participants with diabetes for eGFR (**D**) <30 mL/min/1.73 m^2^, (**E**) <45 mL/min/1.73 m^2^, and (**F**) <60 mL/min/1.73 m^2^.

**Figure 4:**
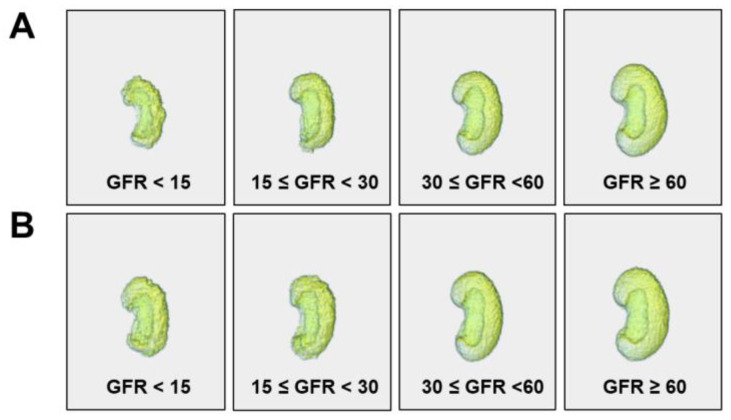
Three-dimensional visualization of representative shapes of the kidney in (**A**) patients without diabetes and (**B**) patients with diabetes. GFR indicates eGFR (mL/min/1.73 m^2^) calculated using the CKD-EPI equation.

ROC analysis showed different diagnostic performances for morphological features (Figure 3). The areas under the ROC curves (AUCs) of the features ranged between 0.51 and 0.86 among the total participants. The overall diagnostic performance was better for eGFR < 30 mL/min/1.73 m^2^ than that for eGFR < 45 mL/min/1.73 m^2^. The SVR showed the highest AUC value (0.83–0.86) for all eGFR thresholds. The kidney volume (estimated by mesh) also had a high AUC (0.81–0.84). The maximum 3D diameter and minor axis length showed better AUCs for CKD than other diameters. The AUC of sphericity ranged from 0.69 to 0.75, while elongation and flatness had relatively poor diagnostic performance (AUC 0.51–0.62). On the other hand, the overall diagnostic performance was relatively lower in patients with diabetes. In particular, the AUCs for diabetic patients with eGFR < 30 or 45 mL/min/1.73 m^2^ were lower than those of the total participants. Nonetheless, the SVR still had relatively higher AUCs compared to other features. A 3D visualization of the representative kidney images is given in Figure 4. The kidney size shrank with worsening GFR, and a markedly rough and bumpy surface was observed in kidneys with low eGFR. These morphological changes were more pronounced in patients with advanced non-diabetic CKD than in those with diabetes.

### 3.4. Determinants of Surface-Area-to-Volume Ratio

Linear regression analysis was performed to identify the determinants of the SVR. Sex, age, body mass index (BMI), eGFR, and hemoglobin level were associated with the SVR in univariable analysis (Table 3).

In multivariable analysis, sex, age, BMI, and eGFR were found to be statistically significant determinants of the SVR. Age (β = 0.0004; *p* = 0.0002) revealed a positive association with the SVR, whereas male sex (β = −0.011; *p* = 0.0001), BMI (β = −0.002; *p* < 0.0001), and eGFR (β = −0.001; *p* < 0.0001) showed negative associations.

## 4. Discussion

In this study, we evaluated the geometrical alterations in the kidney as GFR declines by analyzing the three-dimensional quantitative and objective morphological features. We explored 17 different morphological features derived from CT scans, including kidney volume, axis length, surface area, sphericity, and flatness. As expected from previous studies, kidney volume and length were moderately correlated with kidney function. However, of all the studied morphological features, the SVR showed the highest diagnostic ability for CKD. Other shape features, such as sphericity, compactness, and elongation, may reflect geometrical changes during CKD progression, but their associations with kidney function were moderate to low.

Studies on kidney size and shape in CKD have mainly been based on sonographic measurements determined by radiologists. Numerous studies have observed smaller and thinner kidneys with worsening kidney function [15]. Kidney length and volume measured on kidney US are known to correlate with the GFR. The correlation coefficient between kidney length and function has been reported to be approximately 0.5 [16,17,18]. Kidney volume shows variable correlations with kidney function, depending on the method of volume assessment. Estimating the volume using the ellipsoidal formula yields correlation coefficients similar to those of the kidney length [16,18]. US image acquisition and measurements are highly operator dependent, and an ellipsoid assumption could lead to biased values. We did not manually determine the kidney length and volume, which were calculated mathematically based on 3D voxels without geometrical assumptions. Therefore, in contrast to previous sonographic studies, our methods were objective and were less affected by the bias of the researchers. The kidney size parameters obtained in this study had relatively higher correlations with GFR than those obtained in previous sonographic studies. Notably, the commonly used kidney length is measured along the longest major axis. However, we found that the minor axis length was better correlated with GFR and more diagnostic for CKD. Of the maximum diameters, 2D coronal and 3D diameters are more associated with GFR than the others. Overall, mesh- or voxel-based kidney volumes provided better discrimination for CKD than the diameters. Without geometrical assumptions, summing up slices of axial CT images could provide a more accurate estimation of kidney parenchymal volume, showing a correlation coefficient of 0.62–0.65 [19,20], which is comparable to our results.

In terms of kidney shape, previous studies mainly addressed kidney size parameters without considering kidney shape. Only a few authors have attempted to describe morphological changes by combining simple measures of the kidney. RSI is a kidney shape parameter defined as kidney length (kidney width + kidney thickness) [13]. Nakazato et al. showed that RSI was negatively correlated with age and BMI [13], which is consistent with another study in the Korean population [21]. That is, lower RSI values indicate a plump and thicker kidney in older and obese individuals. However, the relationship between RSI and GFR has yielded conflicting results [22,23]. We evaluated seven different direct shape features (SVR, compactness 1 and 2, sphericity, spherical disproportion, elongation, and flatness). These shape features, except for the SVR, yielded relatively lower correlations with eGFR compared to the kidney size features (volume and diameter). In particular, elongation and flatness were weakly correlated with renal function. Given that RSI roughly represents the ratio of the long to short diameters of the kidney, the definitions of elongation and flatness closely resemble those of RSI. These parameters are based on the assumption of an ellipsoidal shape, which does not accurately reflect the actual bean-shaped morphology of the kidney. Other shape features, such as compactness and sphericity, showed moderate correlations with the eGFR. Kidneys with increased compactness or sphericity are closer in morphology to a perfect sphere. When GFR declines, compactness and sphericity decrease as well, which means that CKD kidneys are far from being spherical, being less round with rough and bumpy surfaces.

Of all the studied morphological features, the SVR had the best diagnostic value. Both the kidney surface area and volume decreased with declining GFR, but the extent of the decrease was much greater in volume than in surface area. This indicates a disproportionate relationship between the kidney volume and surface area during GFR decline. This is one of the novel findings of our study and, thus, SVR could be a potential feature in further applications such as prediction models. In multivariable regression analysis, the SVR increased with lower GFR or BMI but increased with older age. This characteristic of the SVR could be more useful in differentiating CKD from normal aging in the elderly. On the other hand, overall diagnostic values of morphological features were better in patients without diabetes. Diabetic kidney disease is characterized by renal hypertrophy and hyperfiltration during its clinical course [24,25]. The rate of kidney size reduction during CKD progression is relatively lower in diabetic CKD than in non-diabetic CKD [26]. As shown in our 3D visualization of the kidney, morphological changes were more prominent in non-diabetic CKD patients. On the other hand, the AUCs of morphological features were different for eGFR thresholds. This could be related to the differences in the evolution of kidney shape and size according to CKD stages. However, a larger prospective study will be required to confirm this finding.

Although radiomics features include numerous other intensity and texture features, morphological features are robust for image preprocessing and have higher reliability and reproducibility [27]. The morphological features we studied have potential as useful imaging biomarkers for CKD. Beyond the clinical diagnosis of CKD, these image features can be utilized to determine whether impaired kidney function is due to chronic changes in CKD or reversible acute injury. Many decisions about kidney care depend on the chronicity of kidney diseases. Given that kidney shape markers have not been fully explored in CKD, our findings provide valuable evidence for future studies. In particular, given that morphological features reflect chronic structural or pathological changes in CKD, they may be predictive markers for CKD progression.

Our study has some limitations. First, we did not use measured GFR but eGFR, which is derived from a population-based equation and has a bias. Second, our imaging data were collected from a single center, and all participants were Korean. Therefore, a larger multicenter study is warranted to generalize our findings. Third, the extraction of morphological features requires segmentation of the kidney. Automated segmentation is more appropriate than manual segmentation for facilitating its use in clinical settings. Fortunately, recent deep-learning algorithms have been extensively applied to organ or tissue segmentation in medical images [28,29]. In this context, our findings have potential for further clinical applications.

## 5. Conclusions

We presented a quantitative analysis of various 3D shape and size features of the kidney obtained from CT images. Decreased kidney volume, sphericity, axis length, and increased SVR were observed with decreasing GFR. Among the studied morphological features, the SVR showed the highest correlation with eGFR, suggesting that it could be potentially diagnostic for CKD. Our study provides a feature-based quantification and visualization of morphological alterations in CKD kidneys.

## Figures and Tables

**Table 1:** Characteristics of the participants.

Characteristics (*n* = 257)	
Male sex, *n* (%)	143 (55.6%)
Age, median [range], yr	71.0 [19.0–89.0]
BMI, median [IQR], kg/m^2^	24.1 [21.8–26.8]
BSA, mean ± SD, m^2^	1.7 ± 0.2
Comorbidity, *n* (%)	
Diabetes	96 (37.4%)
Hypertension	158 (61.5%)
Coronary artery disease	27 (10.5%)
Serum creatinine, median [IQR], mg/dL	1.2 [0.9–1.6]
Estimated GFR, mean ± SD, mL/min/1.73 m^2^	57.2 ± 26.3
CKD stage (eGFR range), *n* (%)	
Stage 1 (≥90 mL/min/1.73 m^2^)	39 (15.2%)
Stage 2 (60–90 mL/min/1.73 m^2^)	65 (25.3%)
Stage 3 (30–60 mL/min/1.73 m^2^)	114 (44.4%)
Stage 4 (15–30 mL/min/1.73 m^2^)	27 (10.5%)
Stage 5 (<15 mL/min/1.73 m^2^)	12 (4.7%)
Hemoglobin, mean ± SD, g/dL	11.3 ± 2.2
Albumin, median [IQR], g/dL	3.7 [3.2–4.1]

Abbreviations: BMI, body mass index; BSA, body surface area; IQR, interquartile range; SD, standard deviation; GFR, glomerular filtration rate; CKD, chronic kidney disease; eGFR, estimated glomerular filtration rate.

**Table 2:** Correlation of morphological features with eGFR.

Features	Total (*n* = 257)		Patients without Diabetes (*n* = 161)		Patients with Diabetes (*n* = 96)	
	r	*p*-Value	r	*p*-Value	r	*p*-Value
Surface-area-to-volume ratio	−0.747	<0.0001	−0.779	<0.0001	−0.702	<0.0001
Mesh volume	0.686	<0.0001	0.684	<0.0001	0.717	<0.0001
Voxel volume	0.686	<0.0001	0.684	<0.0001	0.712	<0.0001
Minor axis length	0.652	<0.0001	0.672	<0.0001	0.637	<0.0001
Maximum 3D diameter	0.603	<0.0001	0.629	<0.0001	0.593	<0.0001
Maximum 2D diameter (coronal view)	0.605	<0.0001	0.634	<0.0001	0.566	<0.0001
Maximum 2D diameter (axial view)	0.586	<0.0001	0.593	<0.0001	0.603	<0.0001
Maximum 2D diameter (sagittal view)	0.584	<0.0001	0.612	<0.0001	0.58	<0.0001
Surface area	0.583	<0.0001	0.58	<0.0001	0.629	<0.0001
Major axis length	0.551	<0.0001	0.561	<0.0001	0.564	<0.0001
Least axis length	0.454	<0.0001	0.461	<0.0001	0.515	<0.0001
Compactness2	0.439	<0.0001	0.486	<0.0001	0.336	<0.0001
Compactness1	0.437	<0.0001	0.484	<0.0001	0.336	<0.0001
Sphericity	0.435	<0.0001	0.482	<0.0001	0.335	<0.0001
Spherical disproportion	−0.426	<0.0001	−0.471	<0.0001	−0.33	<0.0001
Elongation	0.129	0.039	0.157	0.047	0.076	0.463
Flatness	−0.117	0.062	−0.126	0.112	−0.077	0.456

**Table 3:** Linear regression analysis on the determinants of surface-area-to-volume ratio.

Variables	Univariable		Multivariable	
	Beta Coefficient	*p*-Value	Beta Coefficient	*p*-Value
Sex	−0.012	0.004	−0.011	0.0001
Age	0.001	<0.0001	0.0004	0.0002
BMI	−0.001	0.004	−0.002	<0.0001
Diabetes	−0.001	0.739		
Hypertension	0.005	0.251		
Coronary artery disease	0.012	0.069		
eGFR	−0.001	<0.0001	−0.001	< 0.0001
Hemoglobin	−0.004	<0.0001	0.001	0.107
Albumin	0.005	0.099		

Abbreviations: BMI, body mass index; eGFR, estimated glomerular filtration rate.

## Data Availability

Data supporting the reported results can be obtained on request.

## Supplementary Materials

The following supporting information can be downloaded at: https://www.mdpi.com/article/10.3390/diagnostics13030402/s1, Table S1: The definitions of morphological features; Table S2: Summary of morphological features. Table S3: Correlation of morphological features with eGFR according to sex. Table S4: Correlation of morphological features with eGFR according to BMI. Table S5. Correlation of morphological features with eGFR according to age.
