# Supplementary material for: Changes in CT-Based Morphological Features of the Kidney with Declining Glomerular Filtration Rate in Chronic Kidney Disease
Source: Diagnostics (Basel). 2023 Jan 22;13(3):402. doi: 10.3390/diagnostics13030402 (PMC9914455; doi:10.3390/diagnostics13030402)
Supplement: Supplementary file 1 [file diagnostics-13-00402-s001.zip › diagnostics-2142667-supplementary.pdf]

Supplementary materials

Supplementary Table S1. The definitions of morphological features.

Features	Definition
Mesh volume	Mesh volume is calculated from ROI triangle mesh. V_i means the volume of tetrahedron (a, b, and c are the vertex points of face i) $$V = \left\| \sum_{i=1}^{N_f} V_i \right\|, \quad V_i = \frac{a \cdot (b \times c)}{6}$$
Voxel volume	Voxel volume is estimated from counting voxels. $$V_{voxel} = \left\| \sum_{i=1}^{N_v} V_i \right\|$$
Surface area	Surface area is calculated by summing all the triangular face surface area, $a_i b_i, b_i c_i$ means the edge of mesh triangle. $$A = \left\| \sum_{i=1}^{N_f} A_i \right\|, \quad A_i = \frac{1}{2} \|a_i b_i \times a_i c_i\|$$
Surface area to volume ratio	Surface area to volume ratio is defined as surface area divided by mesh volume. $$Surface\ area\ to\ volume\ ratio = \frac{A}{V}$$
Sphericity	Sphericity measures the roundness of target region. The sphericity ranges from 0 to 1 (perfect sphere). $$Sphericity = \frac{\sqrt[3]{36\pi V^2}}{A}$$
Compactness 1	Compactness 1 measure of how compact the shape of target region. It ranges from 0 to $\frac{1}{6\pi}$ $$Compactness\ 1 = \frac{V}{\sqrt{\pi A^3}}$$
Compactness 2	Compactness 1 has similar to compactness 1, but it is dimensionless, and ranges from 0 to 1. $$Sphericity = 36\pi \frac{V^2}{A^3}$$
Spherical Disproportion	Spherical Disproportion is defined as the ratio of the surface area of the target region to the surface area of a sphere with the same volume as the target region. It is the inverse of sphericity. $$Spherical\ disproportion = \frac{A}{4\pi R^2} = \frac{A}{(36\pi V^2)^{1/3}}$$
Maximum 3D diameter	Maximum 3D diameter is the largest Euclidean distance between target region surface mesh vertices.
Maximum 2D diameter (Slice)	Maximum 2D diameter (Slice) is the largest Euclidean distance between target region surface mesh vertices in the axial plane.

Maximum 2D diameter (Column)	Maximum 2D diameter (Column) is the largest pairwise Euclidean distance between target region surface mesh vertices in the coronal plane.
Maximum 2D diameter (Row)	Maximum 2D diameter (Row) is the largest Euclidean distance between tumor surface mesh vertices in the sagittal plane.
Major Axis Length	Major axis length is the largest axis length of the target region-enclosing ellipsoid. λ_{major} is the largest principal component. $$major\ axis\ length = 4\sqrt{\lambda_{major}}$$
Minor Axis Length	Minor axis length is the second-largest axis length of the target region-enclosing ellipsoid. λ_{minor} is the second-largest principal component. $$minor\ axis\ length = 4\sqrt{\lambda_{minor}}$$
Least Axis Length	Minor axis length is the smallest axis length of the target region-enclosing ellipsoid. λ_{least} is the smallest principal component. $$least\ axis\ length = 4\sqrt{\lambda_{least}}$$
Elongation	Elongation is defined using the ratio of the minor and major principal axis lengths. It ranges from 0 to 1 (circle-like cross section). $$elongation = \sqrt{\frac{\lambda_{minor}}{\lambda_{major}}}$$
Flatness	Flatness is defined using the ratio of the least and major principal axis lengths. It ranges from 0 to 1 (sphere-like). $$flatness = \sqrt{\frac{\lambda_{least}}{\lambda_{major}}}$$

Further details on above features are found elsewhere [1,2].

References

1. Zwanenburg, A.; Vallieres, M.; Abdalah, M.A.; Aerts, H.; Andrearczyk, V.; Apte, A.; Ashrafinia, S.; Bakas, S.; Beukinga, R.J.; Boellaard, R.; et al. The Image Biomarker Standardization Initiative: Standardized Quantitative Radiomics for High-Throughput Image-based Phenotyping. *Radiology* **2020**, *295*, 328-338, doi:10.1148/radiol.2020191145.
2. Pyradiomics community. pyradiomics. Available online: https://pyradiomics.readthedocs.io/ (accessed on November 30, 2022).

Supplementary Table S2. Summary of morphological features.

Features	
Mesh volume (mm^3)	124411.54 ± 45219.88
Voxel volume (mm^3)	124491.55 ± 45233.11
Surface area (mm^2)	24639.04 ± 5763.56
Surface area to volume ratio	0.21 ± 0.03
Sphericity	0.48 ± 0.03
Compactness 1	0.02 ± 0.00
Compactness 2	0.11 ± 0.02
Spherical disproportion	2.09 ± 0.15
Maximum 3D diameter (mm)	107.02 ± 13.08
Maximum 2D diameter (coronal view, mm)	96.65 ± 12.11
Maximum 2D diameter (sagittal view, mm)	102.26 ± 13.07
Maximum 2D diameter (axial view, mm)	64.00 ± 7.86
Major axis length (mm)	99.54 ± 12.18
Minor axis length (mm)	54.49 ± 6.53
Least axis length (mm)	45.10 ± 5.51
Elongation	0.55 ± 0.05
Flatness	0.46 ± 0.05

Supplementary Table S3. Correlation of morphological features with eGFR according to sex.

Features	Males (n=143)		Females (n=114)	
	r	*P*-value	r	*P*-value
Surface volume ratio	-0.719	<0.0001	-0.763	<0.0001
Mesh volume	0.66	<0.0001	0.699	<0.0001
Voxel volume	0.66	<0.0001	0.699	<0.0001
Minor axis length	0.627	<0.0001	0.664	<0.0001
Maximum 3D diameter	0.545	<0.0001	0.633	<0.0001
Maximum 2D diameter (coronal view)	0.582	<0.0001	0.611	<0.0001
Maximum 2D diameter (axial view)	0.537	<0.0001	0.61	<0.0001
Maximum 2D diameter (sagittal view)	0.522	<0.0001	0.615	<0.0001
Surface area	0.539	<0.0001	0.599	<0.0001
Major axis length	0.49	<0.0001	0.575	<0.0001
Least axis length	0.4	<0.0001	0.45	<0.0001
Compactness2	0.566	<0.0001	0.432	<0.0001
Compactness1	0.557	<0.0001	0.428	<0.0001
Sphericity	0.553	<0.0001	0.426	<0.0001
Spherical disproportion	-0.534	<0.0001	-0.414	<0.0001
Elongation	0.11	0.19	0.095	0.314
Flatness	-0.109	0.195	-0.194	0.039

Supplementary Table S4. Correlation of morphological features with eGFR according to BMI.

Features	BMI < 24.11 (n=128)		BMI ≥ 24.11 (n=129)	
	r	*P*-value	r	*P*-value
Surface volume ratio	-0.748	<0.0001	-0.752	<0.0001
Mesh volume	0.697	<0.0001	0.683	<0.0001
Voxel volume	0.697	<0.0001	0.683	<0.0001
Minor axis length	0.662	<0.0001	0.645	<0.0001
Maximum 3D diameter	0.636	<0.0001	0.578	<0.0001
Maximum 2D diameter (coronal view)	0.609	<0.0001	0.607	<0.0001
Maximum 2D diameter (axial view)	0.601	<0.0001	0.578	<0.0001
Maximum 2D diameter (sagittal view)	0.62	<0.0001	0.555	<0.0001
Surface area	0.598	<0.0001	0.576	<0.0001
Major axis length	0.582	<0.0001	0.524	<0.0001
Least axis length	0.451	<0.0001	0.459	<0.0001
Compactness2	0.413	<0.0001	0.5	<0.0001
Compactness1	0.41	<0.0001	0.494	<0.0001
Sphericity	0.408	<0.0001	0.492	<0.0001
Spherical disproportion	-0.399	<0.0001	-0.478	<0.0001
Elongation	0.067	0.453	0.198	0.024
Flatness	-0.162	0.069	-0.089	0.318

Supplementary Table S5. Correlation of morphological features with eGFR according to age.

Features	Age < 71 yr (n=128)		Age ≥ 71 yr (n=129)	
	r	*P*-value	r	*P*-value
Surface volume ratio	-0.749	<0.0001	-0.644	<0.0001
Mesh volume	0.627	<0.0001	0.662	<0.0001
Voxel volume	0.627	<0.0001	0.662	<0.0001
Minor axis length	0.617	<0.0001	0.633	<0.0001
Maximum 3D diameter	0.564	<0.0001	0.599	<0.0001
Maximum 2D diameter (coronal view)	0.581	<0.0001	0.544	<0.0001
Maximum 2D diameter (axial view)	0.564	<0.0001	0.55	<0.0001
Maximum 2D diameter (sagittal view)	0.542	<0.0001	0.587	<0.0001
Surface area	0.54	<0.0001	0.595	<0.0001
Major axis length	0.508	<0.0001	0.564	<0.0001
Least axis length	0.465	<0.0001	0.437	<0.0001
Compactness2	0.444	<0.0001	0.161	0.068
Compactness1	0.448	<0.0001	0.163	0.064
Sphericity	0.449	<0.0001	0.164	0.063
Spherical disproportion	-0.448	<0.0001	-0.165	0.062
Elongation	0.228	0.01	-0.016	0.861
Flatness	0.012	0.891	-0.199	0.024
